# The effectiveness of neuroendoscopic versus non-neuroendoscopic procedures in the treatment of lateral ventricular cysts: a retrospective medical record review study

**DOI:** 10.1186/1471-2377-13-59

**Published:** 2013-06-13

**Authors:** Peng Zhao, Xinsheng Wang, Chuzhong Li, Songbai Gui, Xuyi Zong, Yazhuo Zhang

**Affiliations:** 1 Department of Neurosurgery, Beijing Tiantan Hospital, Capital Medical University, Dongcheng District, Beijing 100050, People’s Republic of China; 2 Beijing Neurosurgical Institute, Capital Medical University, Beijing, 100050, People’s Republic of China

**Keywords:** Neuroendoscope, Lateral ventricle, Arachnoid cyst, Intracranial lesion

## Abstract

**Background:**

The aim of this study was to assess the effectiveness of neuroendoscopy compared with non-neuroendoscopic procedures for treating patients with arachnoid membrane cysts in the lateral ventricles.

**Methods:**

The medical records of 28 patients with arachnoid membrane cysts in the lateral ventricles who were treated with neuroendoscopy and 39 such patients treated with non-neuroendoscopic techniques using classic treatment procedures were reviewed. The neuroendoscopic approach combined craniotomy, corticectomy, lesion resection and cyst ventriculostomy or cyst cisternostomy to restore normal cerebrospinal fluid circulation. The non-neuroendoscopic techniques included craniotomy, corticectomy, and lesion resection performed under a microscope. Clinical outcomes of symptoms and cyst size change on imaging were compared between the two treatment groups during follow-up (range: 1–5 years).

**Results:**

Patients in the neuroendoscopy group had significantly less blood loss (*P* < 0.001) and shorter operative time (*P* < 0.001), better marked improvement in symptoms (64.3% vs. 5.1%, respectively), and a higher total resection rate (92.9% vs. 66.7%; *P* = 0.011) compared with the patients in the non-neuroendoscopy group. In the neuroendoscopy group there was no cyst recurrence whereas in the non-neuroendoscopy group 8 (20.5%) patients had cyst recurrence. However, all patients in the neuroendoscopy group had postoperative transient fever and 8 (28.6%) patients had subdural fluid accumulation which was treated and subsequently resolved during follow-up. These symptoms did not occur in the non-neuroendoscopy group.

**Conclusion:**

We found that neuroendoscopic therapy for arachnoid cysts in the lateral ventricles was more efficacious than non-neuroendoscopic methods. Our results indicate that neuroendoscopy may produce better clinical outcomes than non-neuroendoscopic procedures in treating patients with arachnoid cysts in the lateral ventricles.

## Background

Neuroendoscopy plays an important role in the diagnosis and treatment of a variety of lesions, especially cystic intracranial lesions, both in pediatric and adult patients [1,2]. Cysts are particularly amenable to the neuroendoscopic approach because they are not difficult to aspirate and their walls are easy to ablate or resect [3,4]. This group of space-filling lesions includes colloid, arachnoid, porencephalic, and pineal cysts, as well as Rathke’s cleft cysts, cystic craniopharyngiomas, and malignant tumors with cystic components [2,5]. Neuroendoscopy can be used to excise or reduce the volume of the cyst or as palliative treatment [3,6]. Neuroendoscopy has the advantage of being less invasive than non-neuoendoscopic surgical procedures and is associated with reduced morbidity and mortality and fewer complications, shorter hospital stays, and faster return to work compared with other neurosurgical techniques [2,3,5-8]. Neuroendoscopic techniques can provide simultaneous treatment of hydrocephalus with cyst or resection, thereby avoiding additional invasive procedures [7].

Arachnoid cysts, colloid cysts, and tumors in the lateral ventricles occur frequently and account for about 30% of all intracranial lesions [9-11]. Neuroepithelial cysts in the lateral ventricles are often located in the trigone area, temporal horn, or posterior horn. There have been only a few studies (which included about 10–20 patients) that assessed the use of neuroendoscopy for treating lateral ventricular cysts [12-15]. All patients in this study had arachnoid membrane cysts. These cysts are filled with fluid that is similar to but not the same as cerebrospinal fluid [16]. The cysts are formed from duplication or spitting of the arachnoid layer [16]. Because these cysts are located in the lateral ventricles they block the cerebrospinal fluid circulation route, thereby producing elevated intracranial pressure. Therefore, surgery is required. The purpose of this study was to retrospectively analyze the clinical outcomes of neuroendoscopic surgery versus non-neuroendoscopic surgical methods for patients with arachnoid membrane cysts in the lateral ventricles.

## Methods

### Patients, examinations, clinical data

This was a retrospective medical record review study that was performed according to the Declaration of Helsinki. The study was approved by the Ethical Review Board of Beijing Tiantan Hospital. The medical records of patients who had cysts located in the lateral ventricles and were enrolled from the Neurosurgical Department of Beijing Tiantan Hospital from March 2005 to May 2011 were reviewed. Patients diagnosed with arachnoid membrane cysts in the lateral ventricles and treated surgically either by neuroendoscopy or non-neuroendoscopic methods were selected. The patients were operated on to relieve the symptoms, resect the lesions, and alleviate high intracranial pressure caused by hydrocephalus. Data were included from clinical history, physical examination, CT and/or MRI scans, surgery, pathological findings, and follow-up.

### Surgical method

All operations were performed through the arachnoid-ventricular space. The selection of the appropriate surgical incision was based on the location of the cyst in the lateral ventricle. For neuroendoscopy, a parietal-occipital straight incision was made for cysts in the trigone area of the lateral ventricle, and a temporal straight incision was typically made for cysts in the posterior horn or temporal horn of the lateral ventricle. A temporal straight incision was made at the nearest site to the posterior horn or temporal horn of the lateral ventricle, which resulted in minimal invasive cutting.

The skull in the surgical region was drilled and a 2-cm bone flap was created. Then corticectomy was performed and the ventricle was punctured. A multi-channel rigid endoscope (Rudolf Medical, Fridingen, German) with an external diameter of 8 mm was inserted into the ventricle. To remove the cyst, a bipolar coagulator, biopsy forceps, and microscopic scissors were inserted into the cavity and the cyst was gently pulled out and removed gradually in small pieces. Using this strategy, combined with water-jet resection, the cyst wall was almost totally removed. Care was taken in removing the cysts as they were often adhered to the choroid plexus, and the force of removal could cause intracerebroventricular hemorrhage. Unresectable areas were coagulated to prevent recurrence. The lateral ventricle was irrigated with room temperature saline to remove any remaining unattached cyst wall or other tissue remnants.

According to the clinical symptoms, patients in the non-neuroendoscopy group were treated with classic methods such as microscopic surgery including craniotomy, corticectomy, and lesion resection for cyst resection. The incision was made in the same location as in the neuroendoscopic procedure but the incision had to be long enough to expose the lesion. The burr hole was enlarged to a keyhole craniotomy of about 3–3.5 cm. Under the microscope the membrane was then fenestrated or partially resected.

### Follow-up

Patients were followed up from 1 to 5 years. All patients underwent CT and/or MRI scans 3 months after being discharged. The scans were used to evaluate patient outcome. Improvement was categorized as markedly improved (i.e., symptoms were alleviated and the cyst was either reduced in size or absent); improved (i.e., symptoms were alleviated, but there was no change in cyst size); or no effect (i.e., symptoms were unchanged and the cyst size was the same or, larger, or the number of cysts had increased) [17].

### Statistical analysis

Comparability between the two groups was determined using the Mann–Whitney U test for skewed continuous variables and a chi-square/Fisher’s exact test for categorical variables. Data are presented as the median (interquartile range) for continuous data and numbers (percentages) for categorical data. All statistical assessments were two-sided and evaluated at the 0.05 level of significant difference. Statistical analyses were performed using SPSS 15.0 statistics software (SPSS Inc, Chicago, IL).

## Results

### Patient demographics and disease characteristics

Baseline patient demographics were similar between the neuroendoscopy group (n = 28) and the non-neuroendoscopy group (n = 39) in age and cyst location (all *P* > 0.05) (Table 1). However, there was significant difference in gender and the cyst size between the two groups (*P* < 0.005). For both treatment groups, the most common symptoms at presentation were headache and/or vomiting.

**Table 1:** Patient demographics and baseline characteristics

	**Neuroendoscopy group (n = 28)**	**Non-neuroendoscopy group (n = 39)**	**P-value**
Age (year)^1^	15 (9, 37)	12 (7, 25)	0.712
Gender, n (%)^2^			0.018*
Male	7 (25.0)	21 (53.8)	
Female	21 (75.0)	18 (46.2)	
Location, n (%)^3^			0.810
Left lateral ventricle body	7 (25.0)	12 (30.8)	
Right lateral ventricle body	16 (57.1)	16 (41.0)	
Left lateral ventricle trigone area	2 (7.1)	7 (17.9)	
Right lateral ventricle trigone area	2 (7.1)	4 (10.3)	
Septum pellucidum	1 (3.6)	0 (0.0)	
Cyst size (cm^2^)^1^	6 (2, 6)	6 (6, 12)	0.002*
Symptoms, n (%)^3^			
Headache	16 (57.1)	25 (64.1)	0.617
Vomiting	16 (57.1)	25 (64.1)	0.617
Double vision	2 (7.1)	0 (0.0)	0.171
Intelligence decline	4 (14.3)	2 (5.1)	0.227
Paresis	3 (10.7)	0 (0.0)	0.068

P-values are from ^1 ^Mann–Whitney U tests; ^2 ^chi-square test and ^3 ^Fisher’s exact test.

Data are displayed as ^1^ median (interquartile) and ^2, 3 ^number (percentage) *:there is significant difference (P < 0.005).

**Figure 1:**
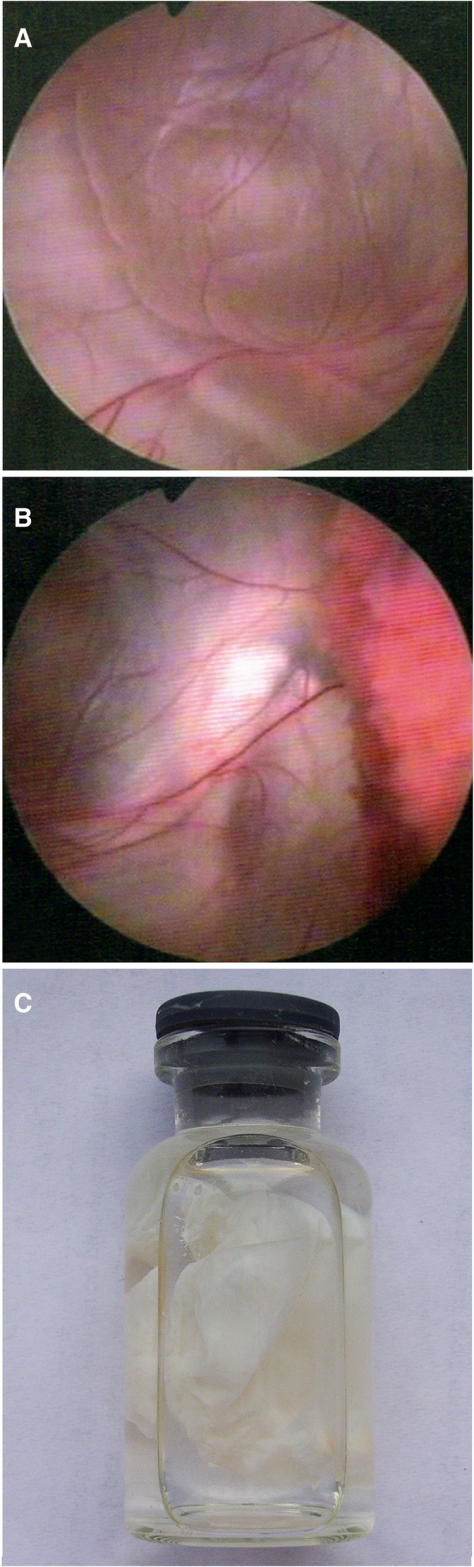
**Endoscopic views of (A) an arachnoid membrane cyst located at the body of the lateral ventricle and (B) adhesion between the intracerebroventricular cyst and the choroid plexus. The cyst was resected from the lateral ventricle (C)**.

**Figure 2:**
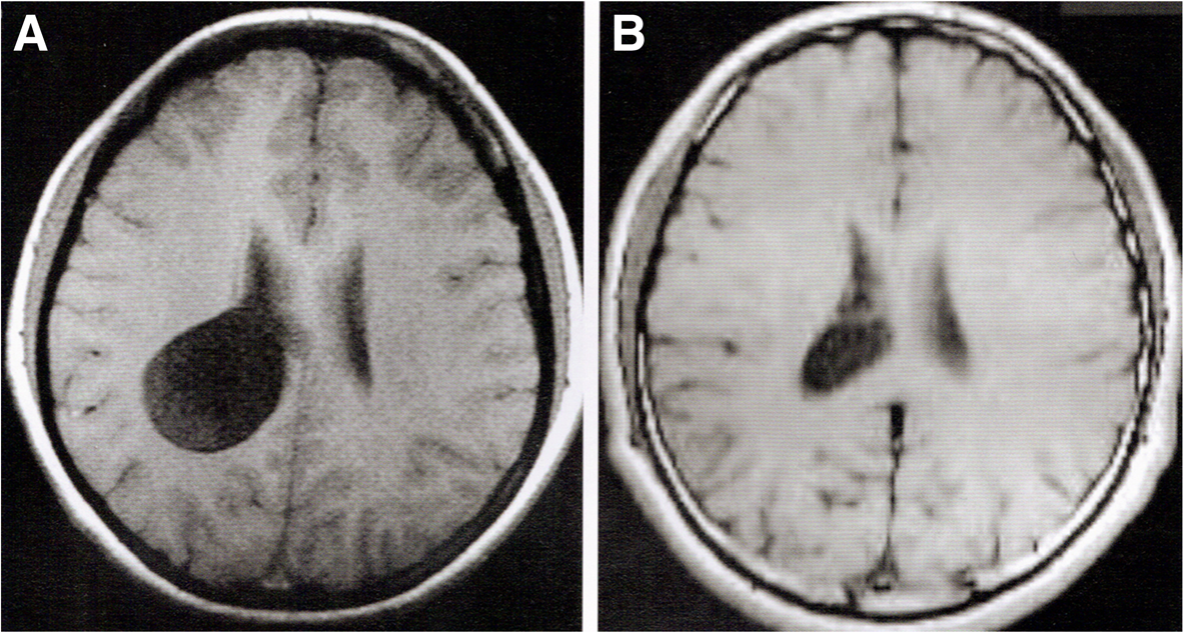
MR image of a cyst was diagnosed in the lateral ventricle (A) before operation and (B) follow-up 3-months after operation.

**Figure 3:**
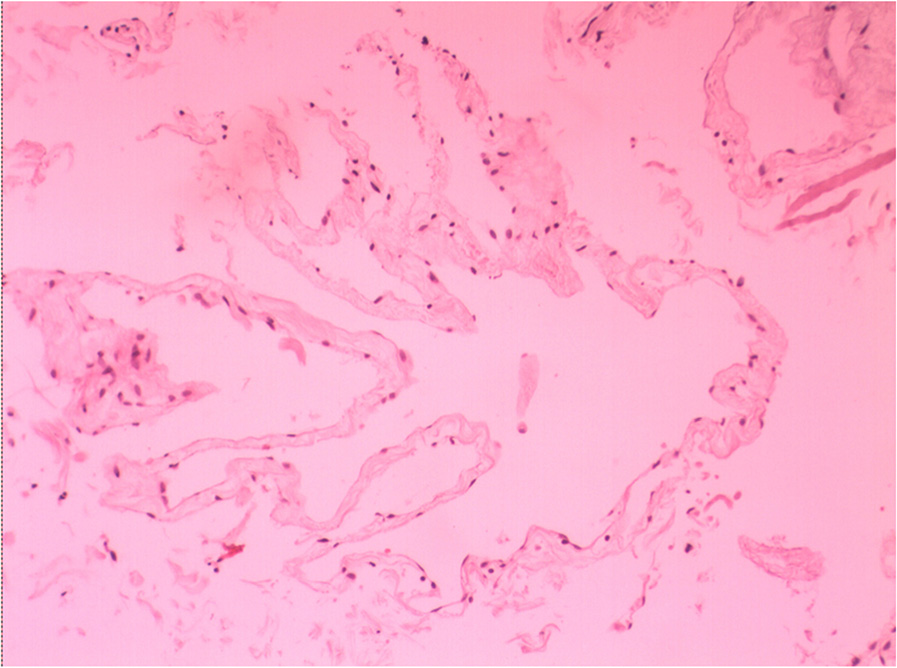
**Photomicrographs showed pathological and immunohistochemical staining of the lesion.** Pathological result demonstrated a cystic wall-like tissue (hematoxylin and eosin; original magnification, 200×).

Figure 1A and B show neuroendoscopic views of a lateral ventricular cyst and Figure 1C shows the resected cyst from a lateral ventricle. Magnetic resonance images of lesions in the lateral ventricle before the operation and 3 months after the operation were obtained regularly (Figure 2). In the pathological examination, cystic wall-like tissue was confirmed (Figure 3).

### Surgical outcomes

Median follow-up duration in both groups was 2 years. In the neuroendoscopy group, 18 (64.3%) and 8 (28.6%) patients had marked improvement and improvement in symptom alleviation, respectively, while in the non-neuroendoscopy group, 2 (5.1%) and 24 (61.5%) patients had marked improvement and improvement in symptom alleviation, respectively. Patients in the neuroendoscopy group had significantly less blood loss and shorter operative time than those in the non-neuroendoscopy group (both *P* < 0.001) (Table 2). But, more patients in the neuroendoscopy group had post-operative fever and subdural fluid accumulation than in the non-neuroendoscopy group (*P* < 0.001). Postoperative fevers in the neuroendoscopy group were non-infectious based on cerebrospinal fluid (CSF) exam and culture, and were most likely the result of intracerebroventricular rinsing during surgery. The fevers resolved after conservative treatment. The subdural fluid was absorbed within 3 months. One patient in the neuroendoscopy group had an intracerebroventricular hemorrhage during surgery which was treated by craniotomy and intracerebroventricular hematoma evacuation. There were no cases of cyst recurrence in the neuroendoscopy group whereas in the non-neuroendoscopy group there were 8 cases (20.5%) of cyst recurrence.

**Table 2:** Outcomes and complications between the 2 groups

	**Neuroendoscopy group (n = 28)**	**Non-neuroendoscopy group (n = 39)**	**P-value**
Duration of follow-up (year)^1^	2 (2, 4)	2 (2, 3)	0.330
Alleviation of symptoms, n (%)^2^			<0.001*
No effect	2 (7.1)	13 (33.3)	
Improvement	8 (28.6)	24 (61.5)	
Marked improvement	18 (64.3)	2 (5.1)	
Total effective resection rate, n (%)^2^	26 (92.9)	26 (66.7)	0.011*
Blood loss (ml)^1^	50 (50, 100)	200 (200, 300)	<0.001*
Hospitalization (day)^1^	10 (12, 15)	10 (10, 40)	0.596
Operative time (hour)^1^	2 (3, 3)	4 (4, 5)	<0.001*
Complication, n (%)^2^			
Fever	28 (100)	0 (0.0)	<0.001*
Subdural fluid	8 (28.6)	0 (0.0)	<0.001*
Subcutaneous fluid	4 (14.3)	1 (2.6)	0.152
Hydrocephalus	0 (0.0)	7 (17.9)	0.036*
Infection	0 (0.0)	1 (2.6)	1.000
Intraventricular hemorrhage	0 (0.0)	3 (5.8)	0.506
Recurrence	0 (0.0)	8 (20.5)	0.017*

*Significant difference between the 2 groups, P < 0.05.

P-values are from ^1 ^Mann–Whitney U test and ^2 ^Chi-square test.

Data are displayed as ^1 ^median (25th , 75th interquartile) and ^2 ^number (percentage).

There was no significant difference of outcomes in the duration of follow-up time between patients treated with neuroendoscopic and non-neuroendoscopic methods (*P* = 0.330) (Table 2). Patients in the neuroendoscopy group compared with patients in the non-neuroendoscopy group had a higher total resection rate (92.9% vs. 66.7%, respectively; *P* =0.011).

In the neuroendoscopy group, there was one case in which the symptoms were not relieved following cyst resection 6 months after the operation. This patient underwent a ventriculoperitoneal shunt operation. There were no deaths in either treatment group.

## Discussion

Prior studies have indicated that neuroendoscopy is a safe and effective method for treating patients with intracranial cysts [1,18-22]. However, only a few studies have investigated the use of neuroendoscopy for treating cysts located in the lateral ventricles [12-15]. In this study, patients treated with neuroendoscopy had generally better clinical outcomes than patients treated with non-neuroendoscopic procedures. The total resection rate was higher for neuroendoscopy versus non-neuroendoscopy (*P* <0.05). We attribute this to the endoscopic approach improving visualization in tight spaces, especially within the lateral ventricle, and to the degree of freedom in creating surgical corridors, etc. Neuroendoscopy was also associated with less operation-related blood loss, shorter surgical time, and greater improvement in symptoms compared with non-neuroendoscopy. Moreover, the proportion of patients with post-operative hydrocephalus was higher in the non-neuroendoscopy group.

Neuroendoscopy is an important option for the treatment of intracranial cysts. Microsurgical approaches that include craniotomy and fenestration, and cystoperitoneal shunting are valuable techniques, however, endoscopy which allows closer direct cyst or tumor visualization, is less invasive, and decreases severe complications associated with other surgical methods. These properties are particularly important in treating young patients with different types of benign tumors [1,21]. Neuroendoscopy has been recommended as the first choice of therapy for treating intracranial cysts [8,18,21-23], including cysts located in the lateral ventricles [12,24]. It has been recommended that when the manipulations are done through the endoscope that neuroendoscopy is best performed on deep-seated cysts such as those in the lateral ventricles as it is relatively less traumatic to the brain parenchyma and provides good visualization [24]. Also, once the endoscope is fixed in position, instruments and optics can be changed readily without damage to brain structures along the approach. Also, endoscopic cyst fenestration from the lateral or third ventricle based on cyst extension presents no risk with regard to damaging the deep incisural and quadrigeminal veins [25]. A disadvantage of this method is the limited range of motion and the size of the instruments that fit into the working channel of the endoscope [24]. Our study supports the idea of removing cysts located in the lateral ventricles by neuroendoscopy where cyst removal is performed through the endoscope itself. In our study, no patients treated with neuroendoscopy had cyst recurrence while cysts recurred in 20.5% of patients treated by non-neuroendoscopic techniques. Gangemi et al. reported that neuroendoscopy was associated with a greater frequency of cyst recurrence compared with other surgical techniques [25]. Our results differed. The reason for this difference is not clear. We used rigid neuroendoscopy to resect arachnoid membrane cysts in the lateral ventricles. Rigid neuroendoscopy was also reported to have been used successfully for the treatment of cerebral aneurysms [26] and colloid cysts [8]. Successful use of flexible neuroendoscopy has been reported for the treatment of supracellar arachnoid cysts [27] and shunt malfunction [28].

Our findings are mostly similar to those of other studies that compared the efficacy and safety of neuroendoscopy to microsurgical resection in treating intraventricular cysts. Several studies reported that neuroendoscopy resulted in less morbidity and shorter hospital stay for adults and children, and more rapid return of the patients to work [5-8,15,18]. King and colleagues reported their experiences with endoscopic resection of colloid cysts. The results showed that the average hospital stay following treatment of colloid cysts of the lateral and third ventricles by neuroendoscopy was 2.3 days compared to 5 days following craniotomy [8]. However, in our study, the length of hospital stay was the same for both the neuroendoscopy and non-neuroendoscopy groups (10 days).

The use of neuroendoscopy to treat intraventricular cysts is also associated with marked improvement in symptoms that include headache, nausea, vomiting, double vision, and paresis [8,9,15]. We found that the majority of patients (64.3%) treated with neuroendoscopy had marked improvement in their symptoms, whereas only 5.1% of patients treated with a non-neuroendoscopy method had marked symptom improvement.

Many complications have been reported for non-neuroendoscopy and neuroendoscopy cyst treatment options. Complications of fenestration/resection and shunting procedures include meningitis, hemiparesis, oculomotor palsy, subdural hematomas, new epileptic seizure, hemorrhages, transient diabetes insipidus, psychosyndrome, and death [22,23]. In our study, neuroendoscopy compared with non-neuroendoscopy was associated with a greater proportion of patients having post-operative fever or subdural fluid accumulation. But these complications soon resolved.

Limitations of this study include its retrospective design and small sample size. Larger prospective studies are necessary to more fully compare the different methods for treating lateral ventricular cysts. Median follow-up duration was only 2 years; a longer follow-up period is needed for more complete assessment. This study also did not evaluate potential differences in the functional impairment of patients following neuroendoscopy versus non-neuroendoscopic surgery. This is of interest since a large trial (N =714) that assessed the clinical outcomes and quality-of-life of patients with ventricular and paraventricular cysts (12.7% of whom had lateral ventricular cysts) found that the Karnofsky performance score significantly improved with neuroendoscopy (from 80 to 90; *P* < 0.0001) [9].

## Conclusion

Minimally invasive neurosurgery is the standard technique for treating a number of neurological disorders. Neuroendoscopy is effective and relatively safe for treating patients with interventricular lesions. This is one of the first studies specifically designed to compare the efficacy and safety of neuroendoscopy with non-neuroendoscopic procedures for treating cysts located in the lateral ventricles. Our findings suggest that neuroendoscopy is effective for treating lateral ventricular cysts and in general resulted in better symptom relief and clinical outcomes than non-neuroendoscopic strategies.

### Consent

Written informed consent was obtained from the patient for publication of this report and any accompanying images.

## Competing interest

All authors have no competing interests.

## Authors’ contributions

YZ: guarantor of integrity of the entire study; study design; clinical studies; experimental studies; manuscript review. PZ: literature research; data acquisition; manuscript preparation; manuscript editing. XZ: study concepts; clinical studies; experimental studies. XW: definition of intellectual content; clinical studies; experimental studies. CL: data acquisition; data analysis. SG: data acquisition; statistical analysis. All authors have read and approved the final manuscript.

## Pre-publication history

The pre-publication history for this paper can be accessed here:

http://www.biomedcentral.com/1471-2377/13/59/prepub
